# Convergence Analysis of Rust Fungi and Anther Smuts Reveals Their Common Molecular Adaptation to a Phytoparasitic Lifestyle

**DOI:** 10.3389/fgene.2022.863617

**Published:** 2022-04-08

**Authors:** Xianzhen Zhou, Dan Yu, Zhimin Cao

**Affiliations:** College of Forestry, Northwest A&F University, Yangling, China

**Keywords:** rust fungi, anther smuts, convergent evolution, amino acid substitution, positive selection, gene gain or loss

## Abstract

Convergent evolution between distantly related taxa often mirrors adaptation to similar environments. Rust fungi and anther smuts, which belong to different classes in Pucciniomycotina, have independently evolved a phytoparasitic lifestyle, representing an example of convergent evolution in the fungal kingdom. To investigate their adaptations and the genetic bases underlying their phytoparasitic lifestyles, we performed genome-wide convergence analysis of amino acid substitutions, evolutionary rates, and gene gains and losses. Convergent substitutions were detected in *ATPeV0D* and *RP-S27Ae*, two genes important for the generation of turgor pressure and ribosomal biosynthesis, respectively. A total of 51 positively selected genes were identified, including eight genes associated with translation and three genes related to the secretion pathway. In addition, rust fungi and anther smuts contained more proteins associated with oligopeptide transporters and vacuolar proteases than did other fungi. For rust fungi and anther smuts, these forms of convergence suggest four adaptive mechanisms for a phytoparasitic lifestyle: 1) reducing the metabolic demand for hyphal growth and penetration at the pre-penetration stage, 2) maintaining the efficiency of protein synthesis during colonization, 3) ensuring the normal secretion of rapidly evolving secreted proteins, and 4) improving the capacity for oligopeptide metabolism. Our results are the first to shed light on the genetic convergence mechanisms and molecular adaptation underlying phytoparasitic lifestyles in fungi.

## Introduction

As heterotrophs, many fungi are parasitic, where they develop a feeding relationship with other organisms (Zelmer, 1998). To obtain nourishment from hosts, parasitic fungi also need to penetrate the outer defenses of hosts and evade the immune response, often having harmful consequences for the hosts (Ewald, 1983). The spectrum of hosts ranges from plants and animals to fungi (Viterbo and Horwitz, 2010; Sharon and Shlezinger, 2013; Köhler et al., 2015), which has been implicated as one of the reasons for the high species diversity of fungi. Most parasitic fungi are parasites of plants and can cause severe damage to a wide range of agricultural crops, tree species used for wood production, and herbaceous plants with ornamental value (Pinon and Frey, 2005; Fontaine et al., 2013; Gramacho et al., 2013). To efficiently control phytoparasitic fungi, research on the genetic mechanisms underlying the phytoparasitic lifestyle is necessary.

Comparative genomics improved our understanding of how phytoparasitic lifestyles evolved. Phytoparasitic fungi possess very plastic and dynamic genomes because of the presence of many transposable elements (TEs) (Möller and Stukenbrock, 2017). This genomic environment has given a competitive advantage to phytoparasitic fungi that need to adapt constantly to the plant immune system (Raffaele and Kamoun, 2012). For phytoparasitic fungi, the expanded genes mainly encode lytic enzymes and putative transporters, while contracted gene families are involved in digesting plant cell walls (Powell et al., 2008). Transcriptomics technology has also helped reveal how phytoparasitic fungi change their gene expression upon host colonization. In most transcriptomics studies, a large proportion of genes were upregulated upon host colonization relative to ribosome-associated proteins, effectors, secreted enzymes, and membrane transporters. These responses involve forming infection structures, avoiding recognition, manipulating host defense, and absorbing nutrients from the host plant (van der Does and Rep, 2017). Although some molecular mechanisms underlying the phytoparasitic lifestyle have been revealed by comparative genomics and transcriptomics technology, how fungi adapt to a phytoparasitic lifestyle remains unclear.

Convergent evolution refers to the process in which species that are not closely related independently evolve similar traits for adaptation to similar selective pressures (Christin et al., 2010; Ghiselin, 2016). Phenotypic convergence often results from genetic convergence at multiple levels. Most studies fulfill the predominant criterion for inferring convergent molecular evolution, which is that the amino acid position of a protein changes from a different ancestral amino acid to the same descendant amino acid along independent evolutionary lineages (Zhang and Kumar, 1997). Examples include echolocation in bats and toothed whales (Parker et al., 2013), the bamboo diet of giant and red pandas (Hu et al., 2017), and the ability to live in marginal environments in mangroves (Xu et al., 2017). According to a more relaxed definition, convergence could also mean copy number or evolutionary rate changes in the same genes (Denoeud et al., 2014; Maria et al., 2016). Overall, convergence analyses provide more opportunities to reveal genetic mechanisms of adaptive evolution.

There is a great diversity of lifestyles among fungi in Pucciniomycotina, including phytoparasites, mycoparasites, entomoparasites, and saprobes (Aime et al., 2014). Two distantly related fungi, rust fungi in the order Pucciniales and anther smut fungi from Microbotryales, are the primary representatives of phytoparasitic fungi in Pucciniomycotina, causing a serious threat to agriculture and forestry worldwide (Aime et al., 2017; Begerow et al., 2017). In contrast, almost all the remaining members of Pucciniomycotina are nonphytoparasitic fungi (Aime et al., 2014). Phytoparasitic lifestyles have evolved independently in rust fungi and anther smuts, making them an ideal model of convergent evolution. To identify the genetic mechanism underlying convergence between these two distantly related phytoparasitic fungi, we performed analyses with three different perspectives: 1) the genes containing convergent amino acid substitutions between rust fungi and anther smuts, 2) the genes subjected to positive selection during the evolutionary process of rust fungi and anther smuts, and 3) convergent gene gain and loss in rust fungi and anther smuts. Our findings may help elucidate the adaptive mechanism associated with phytoparasitism in rust fungi and anther smuts. These findings might also facilitate the development of novel antifungal strategies for rust fungi and anther smuts.

## Materials and Methods

### Classification of Selected Fungi

The flow chart presented in Supplementary Figure S1 illustrates the main steps in our study. A total of 19 fungi were selected, including six rust fungi, three anther smuts, seven nonphytoparasitic fungi in Pucciniomycotina, and three nonbiotrophic fungi in Ustilaginomycotina. Among them, *Austropuccinia psidii* APSI1 (Aps), *Cronartium quercuum* f. sp. *fusiforme* G11 version 1.0 (Cqf) (Pendleton et al., 2014), *Hemileia vastatrix* XXXIII (Hvs), *Melampsora larici-populina* 98AG31 version 1.0 (Mlp) (Duplessis et al., 2011), *Puccinia striiformis* f. sp. *tritici* PST-130 (Pst) (Cantu et al., 2011), and *Uromyces viciae-fabae* I2 (Uvf) were selected as analytical data for representative rust fungi. *Microbotryum intermedium* 1389 BM 12 12 (Min) (Branco et al., 2017), *Microbotryum lychnidis-dioicae* p1A1 Lamole (Mld) (Perlin et al., 2015), and *Microbotryum silenes-dioicae* 1303 (Msd) were selected as phytoparasitic fungi for convergence analyses. The following nonphytoparasitic fungi were included for comparison: *Cystobasidiopsis lactophilus* JCM 7595 (Cla), *Cystobasidium minutum* MCA 4210 version 1.0 (Cmi) (Camiolo et al., 2019), *Erythrobasidium yunnanense* JCM 10687 (Eyu), *Glaciozyma antarctica* PI12 (Gan), *Leucosporidiella creatinivora* 62-1032 version 1.0 (Lcr), (Mondo et al., 2017), *Rhodotorula graminis* strain WP1 version 1.1 (Rgr) (Firrincieli et al., 2015), and *Sporobolomyces roseus* SR19 (Sro). Finally, *Acaromyces ingoldii* MCA 4198 (Ain) (Kijpornyongpan et al., 2018), *Malassezia globosa* CBS 7966 (Mgl) (Xu et al., 2007), and *Pseudozyma antarctica* T-34 (Pan) (Morita et al., 2013), which belong to three different classes in Ustilaginomycotina, were selected as the outgroup taxa.

The data of 11 selected fungi (Cqf, Mlp, Pst, Min, Mld, Cmi, Lcr, Rgr, Ain, Mgl, and Pan) are publicly available in the JGI MycoCosm portal (https://mycocosm.jgi.doe.gov/mycocosm/home). The protein and coding sequences (CDSs) of Msd were downloaded from the NCBI database. Genome assemblies without gene annotation for an additional seven fungi (Aps, Hvs, Uvf, Cla, Eyu, Gan, and Sro) publicly available in the NCBI database were downloaded. Then, protein-coding genes were predicted by BRAKER2 (Hoff et al., 2019). Detailed information for the selected fungi is available in Supplementary Table S1.

### Building Orthologous Gene Groups and Phylogenomic Tree Construction

All amino acid sequences of the 19 fungi were analyzed to obtain orthologous groups by OrthoFinder with default parameters (Emms and Kelly, 2016). To determine the phylogenetic relationships between the selected fungi, we conducted a phylogenetic analysis using all the single-copy genes. Multiple sequence alignments of single-copy orthologous genes were performed using MAFFT (Katoh and Standley, 2013), and the gaps were removed using trimAl (Capella-Gutiérrez et al., 2009). Next, the genes were concatenated to generate a supergene sequence. The best-fitting model for phylogenetic analysis was estimated using ModelTest-NG (Darriba et al., 2020). With the input of these 19 supergene sequences, a phylogenetic tree was constructed using RAxML-NG software with 1,000 bootstrap replicates (Kozlov et al., 2019).

### Convergence Analysis

**FIGURE 1:**
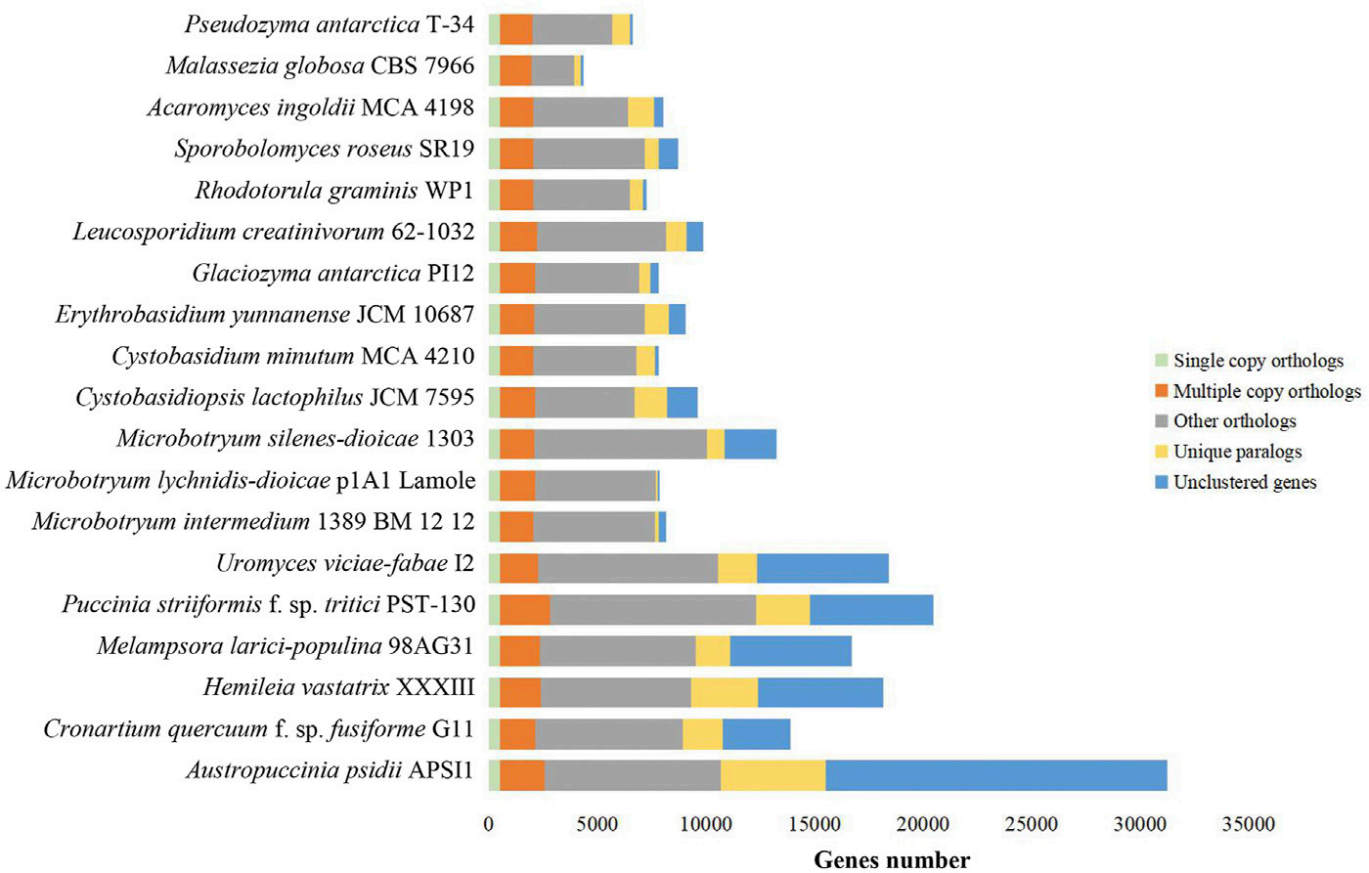
Orthologous groups for 19 selected fungi. Bars are subdivided to represent five different types of orthologs’ relationships. “Single_copy_orthologs” indicates one to one gene among all selected fungi. “Multiple_copy_orthologs” indicates other conserved genes present in multiple copies among all selected fungi. “Other_orthologs” indicates those genes present in at least two fungi, but not all fungi. “Unique_paralogs” indicates species-specific duplicated genes. “Unclustered_genes” indicates no homologous relationship between one and any other taxa.

Only single-copy genes were utilized to identify convergent amino acid sites. First, ancestral states were reconstructed using CodeML. We set node “a” as the ancestral state of rust fungi, node “b” as the most recent common ancestor (MRCA) of rust fungi and other nonphytoparasitic fungi, node “c” as the ancestral state of anther smuts, and node “d” as the MRCA of anther smuts and other nonphytoparasitic fungi (Figure 1). We identified convergent amino acid sites between rust fungi and anther smuts with the following rules: 1) the amino acid residues of node a and node c were identical, 2) amino acid changes were inferred to have occurred between node a and node b, and 3) amino acid changes were inferred to have occurred between node c and node d. Noteworthily, detection of false and random convergence events may result from limited taxon sampling.

Considering that conserved sites permit a more accurate inference of the ancestral state to avoid false convergence (Xu et al., 2017), the convergent amino acid sites with high support were selected with the following rules: 1) the amino acid residues of all rust fungi and anther smuts were identical, 2) there was a significant difference in the usage of amino acids between phytoparasitic and nonphytoparasitic fungi (*p* values less than 0.0005, calculated by Fisher’s exact test), and 3) more than five nonphytoparasitic fungi in Pucciniomycotina and more than two nonphytoparasitic fungi in Ustilaginomycotina tended to use the same amino acid. In general, the ancestral states of node a and node c should be more accurate when the first rule is met. The second and third rules were used to improve the accuracy of the ancestral states of node b and node d.

To filter out noise resulting from chance amino acid substitutions, we then performed a Poisson test to verify whether the observed number of highly supported convergent sites of each gene was significantly greater than the number expected with random substitution under the JTT-fgene model, following the method of Zou and Zhang (2015) (conv_cal, https://github.com/ztzou/conv_cal). The Benjamini–Hochberg method was used to correct multiple testing (*q* < 0.05).

### Testing for Positive Selection

The aligned protein-coding sequences and the species tree obtained from the phylogenetic analysis were used to detect positive selection using the branch-site model in the CodeML program of PAML (Yang, 1997). For every single-copy gene, the branch-site model (model = 2 and NSsites = 2) of codon evolution was used. To identify genes under adaptive evolution related to phytoparasitism, the ancestral branch of rust fungi and the ancestral branch of anther smuts were designated as foreground branches, and the other branches were designated as background branches. We compared the model (which allowed sites to be under positive selection; fix_omega = 0) with the null model (in which sites may evolve neutrally or under purifying selection; fix_omega = 1 and omega = 1) with likelihood ratio tests (LRTs) performed by the CodeML program in PAML. The LRT statistics were evaluated by calculating the 2ΔLnL between the abovementioned models. The significance of LRT statistics was determined by a *χ* ^2^ distribution, and *p* values were adjusted by following the Benjamini–Hochberg procedure. The alternative model was considered to be significantly better than the null model when *q* < 0.05.

### Analyses of Gene Gain and Gene Loss

MCMCTree analysis was applied to estimate divergence times among the 19 fungi (Yang, 1997). According to the TimeTree database (http://www.timetree.org/), ancestral Basidiomycota fungi diverged into Ustilaginomycotina and Pucciniomycotina 489 million years ago (MYA), while the MRCA of *Puccinia* and *Melampsora* diverged 114.5 MYA (Kumar et al., 2017). Using these two fossil calibration points, we estimated the divergence times of the 19 selected fungi based on all single-copy genes. After acquiring this ultrametric tree, orthologous gene group expansion and contraction were detected by CAFE v5.0 (Mendes et al., 2020). Only the multicopy gene group that contained at least one protein in no fewer than eight species was chosen for analysis. Then, the *p-*value was calculated for each orthologous group, and orthologous groups with *p* values less than 0.05 were considered to have accelerated rates of expansion and contraction. The groups that had specifically expanded and contracted in rust fungi and anther smuts were considered candidate gene groups. We also performed a manual check to identify genes specifically missing or present in selected rust fungi and anther smuts.

## Results

### Orthologous Groups, Single-Copy Genes, and the Phylogeny

**FIGURE 2:**
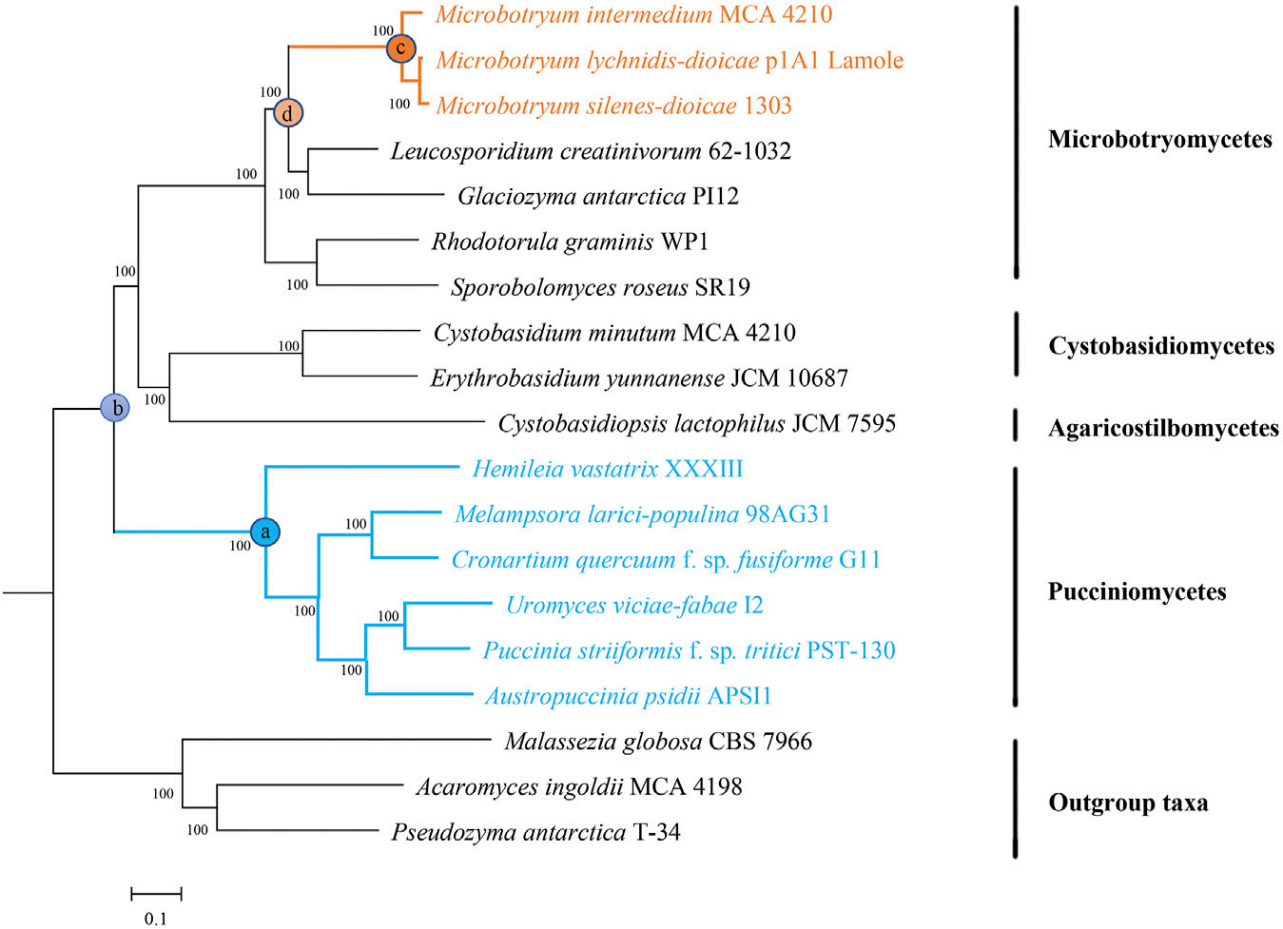
Genome-wide phylogenetic tree of 19 selected fungi. All single-copy genes were used to infer the phylogenomic tree topology using RAxML-NG. Numbers near the nodes are bootstrap support values. Anther smuts and rust fungi are indicated in orange and blue, respectively.

To study whether convergent evolution occurs in rust fungi and anther smuts, we selected 19 basidiomycetous fungi, including 16 fungi in Pucciniomycotina (six rust fungi, three anther smuts, and seven nonphytoparasitic fungi) and three nonphytoparasitic fungi in Ustilaginomycotina, for evolutionary analysis. We first identified all the orthologous gene groups in these fungi. In total, 19,479 orthologous gene groups were identified, including 530 single-copy orthologous gene groups and 1,276 multi-copy orthologous gene groups across all selected fungi (Supplementary Tables S2, S3). Consistent with the findings of previous studies (Cuomo et al., 2017), it is notable that rust fungi have very different gene contents from other selected fungi, harboring a larger number of unique paralogs (2,592, on average) and unclustered genes (7,004, on average) (Figure 2; Supplementary Table S2).

To identify the genomic signatures of convergent evolution between rust fungi and anther smuts, a maximum likelihood phylogeny was reconstructed on the basis of all the proteins encoded by the single-copy genes. The constructed genome-wide phylogenetic tree showed reasonable phylogenetic relationships. Within Pucciniomycotina, three anther smuts were clustered together with other Microbotryomycetes fungi. Compared with other selected Pucciniomycotina fungi, six rust fungi have occupied a basal position (Figure 1).

### Convergent Signatures Between Rust Fungi and Anther Smuts

All 530 single-copy genes were used for genomic convergence analyses. Four hundred forty-four genes with 2,150 convergent amino acid substitutions between rust fungi and anther smuts were identified based on our initial criteria. Because genomic signatures of molecular convergence are generally not strong (Zou and Zhang, 2015), we surmised that random and false convergence events occurred frequently. Seventy-six genes with 84 highly supported sites that fit further criteria were chosen for the Poisson test. Overall, the Poisson test revealed that only two genes (OG0002989 and OG0003182) exhibited stronger signatures of convergent amino acid substitution than expected by chance (*q* < 0.05) (Supplementary Table S4).

**FIGURE 3:**
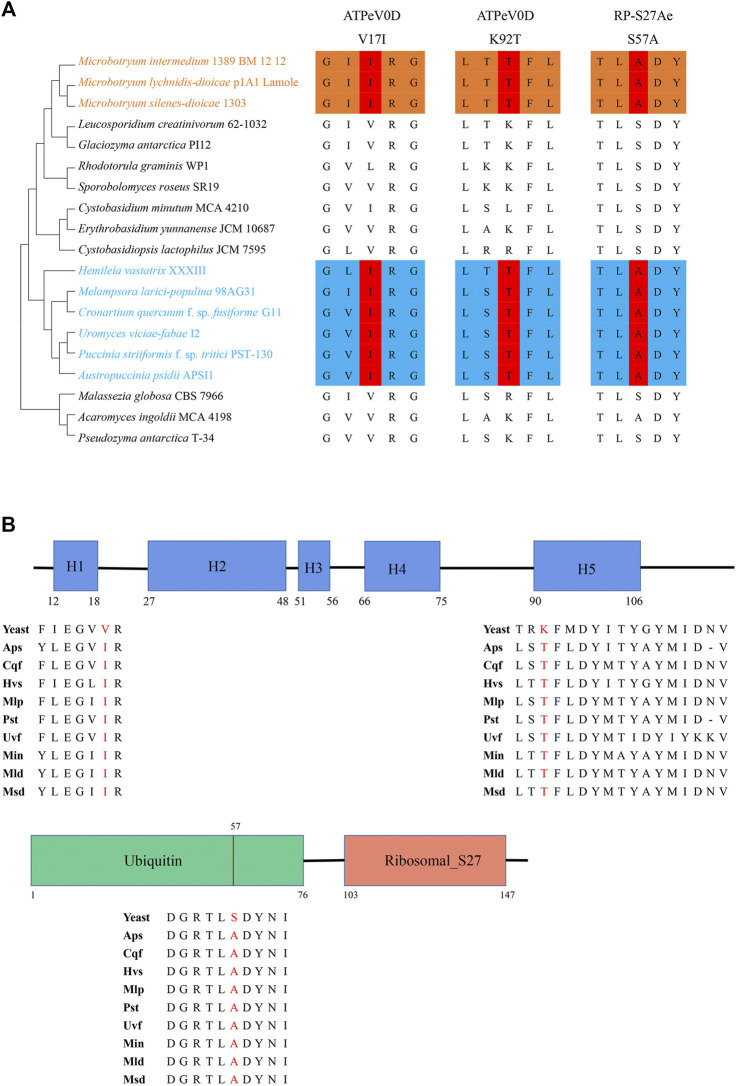
Two convergent genes. **(A)** Convergent amino acid substitutions in ATPeV0D and RP-S27Ae among the 19 fungi. The number above the convergent site denotes the amino acid position. **(B)** Structural domains of ATPeV0D and RP-S27Ae and the locations of three convergent amino acid sites. The structural domain of ATPeV0D was annotated based on the VMA6 protein in *Saccharomyces cerevisiae*, and that of RP-S27Ae was annotated based on the RPS31 protein in *S. cerevisiae*.

These two convergent genes encode V-type H^+^-transporting ATPase subunit d (*ATPeV0D*) and ubiquitin-small subunit ribosomal protein S27Ae (*RP-S27Ae*). For ATPeV0D, two convergent substitutions occurred at site 17 (valine in nonphytoparasitic fungi to isoleucine in phytoparasitic fungi) and site 92 (lysine in nonphytoparasitic fungi to threonine in phytoparasitic fungi). These two sites are located in helix 1 and helix 5, respectively. Helix 1 is required for ATPase subunit assembly, and helix 5 contributes to the translocation of protons (Owegi et al., 2006). For RP-S27Ae, one convergent substitution occurred at site 57 (serine in nonphytoparasitic fungi to alanine in phytoparasitic fungi). This site is a phosphorylation site in the ubiquitin domain that regulates ubiquitin homeostasis (Figure 3) (Lee et al., 2017).

### Positive Selection Signals in Rust Fungi and Anther Smuts

Across all 530 gene orthologues, we found convergent signals of positive selection in 51 genes during the evolution of rust fungi and anther smuts (Supplementary Table S5). To further characterize the functions of positively selected genes, we performed functional annotation with the Kyoto Encyclopaedia of Genes and Genomes (KEGG) database. In total, 43 of the 51 genes had KEGG annotations. Among them, 25 genes were associated with 27 pathways (Table 1). The genes were associated with very diverse pathways, including four main categories: metabolism, genetic information processing, environmental information processing, and cellular processes. There were eight genes in translation pathways (pathway ids: 00970, 03008, 03010, 03013, and 03015), which was the most abundant category. In addition, we found three positively selected genes associated with secretory pathways. They included a protein transport protein (*Sec24*, OG0003606), calnexin (*CANX*, OG0003436), and dolichyl-phosphate-mannose-protein mannosyltransferase (*POMT*, OG0002993). However, genes associated with other functions were relatively small in number.

**TABLE 1:** KEGG pathways of positively selected genes.

Categories	Map.ID and Map.Title	Orthogroup
Metabolism	00190 Oxidative phosphorylation	OG0003237
	00240 Pyrimidine metabolism	OG0003667
	00270 Cysteine and methionine metabolism	OG0003599
	00310 Lysine degradation	OG0003745
	00480 Glutathione metabolism	OG0003599
	00514 Other types of O-glycan biosynthesis	OG0002993
	00515 Mannose type O-glycan biosynthesis	OG0002993
	00562 Inositol phosphate metabolism	OG0003222
	00640 Propanoate metabolism	OG0003355
Genetic Information Processing	00970 Aminoacyl-tRNA biosynthesis	OG0003372, OG0003343
	03008 Ribosome biogenesis in eukaryotes	OG0003110, OG0003348
	03010 Ribosome	OG0002961, OG0003082
	03013 Nucleocytoplasmic transport	OG0003348
	03015 mRNA surveillance pathway	OG0003232
	03018 RNA degradation	OG0003006
	03040 Spliceosome	OG0003532
	03410 Base excision repair	OG0003790
	03430 Mismatch repair	OG0003203
	04141 Protein processing in endoplasmic reticulum	OG0003606, OG0003436
Environmental Information Processing	02010 ABC transporters	OG0003065
	04070 Phosphatidylinositol signaling system	OG0003222
Cellular Processes	04111 Cell cycle—yeast	OG0003471
	04113 Meiosis—yeast	OG0003471
	04138 Autophagy—yeast	OG0003222
	04144 Endocytosis	OG0003073
	04145 Phagosome	OG0003172, OG0003436
	04146 Peroxisome	OG0003121

### Expansion and Contraction of Orthologous Gene Groups in Rust Fungi and Anther Smuts

To identify convergent gene gain or loss in rust fungi and anther smuts, an analysis was performed in CAFÉ. First, a clock-calibrated phylogenetic tree was constructed based on the concatenation of all single-copy genes (Supplementary Figure S2). A total of 4,559 orthologous gene groups were used for CAFÉ analysis by filtering (Supplementary Table S6). There were a few orthologous gene groups that showed a convergent pattern of expansion or contraction between rust fungi and anther smuts. Only two gene groups were specifically expanded in the selected rust fungi and anther smuts. One orthologous group (OG0000061) was annotated as oligopeptide transporters (OPTs), and the other group (OG0000075) was associated with vacuolar protease (Table 2). However, there were no orthologous gene groups that showed a convergent pattern of contraction. Furthermore, no genes were specifically absent or present in rust fungi and anther smuts.

**TABLE 2:** Two expanded orthologous groups in rust fungi and anther smuts.

Orthogroup	Functional annotation	Rust fungi						Anther smuts			Nonphytoparasitic fungi									
		Aps	Cqf	Hvs	Mlp	Pst	Uvf	Min	Mld	Msd	Cla	Cmi	Eyn	Gan	Lcr	Rgr	Sro	Ain	Mgl	Pan
OG0000061	OPT transporter	7	12	9	9	12	6	12	11	10	0	0	0	0	4	4	3	4	0	2
OG0000075	Vacuolar protease	4	6	7	6	9	5	17	14	10	2	0	0	2	3	1	2	0	0	1

## Discussion

By resolving the phylogeny of selected taxa, we clearly revealed that phytoparasitic lifestyles have evolved independently in rust fungi and anther smuts. Node b is the common ancestor of rust fungi and anther smuts. If rust fungi and anther smuts evolved a phytoparasitic lifestyle at the same time, all (or at least most) of the species arising from node b should be phytoparasitic, which is not consistent with the data (Figure 1). The phylogeny reconstructed in this study does not show the same topology as that in some previous studies in which Agaricostilbomycetes and Cystobasidiomycetes were sister to both Pucciniomycetes and Microbotryomycetes (Schell et al., 2011; Wang et al., 2015; Zhao et al., 2017). This is probably caused by the difference in datasets employed. Our results were inferred from the concatenation of single-copy genes, while previous studies were performed using one or several specific genes (ITS, RPB1, RPB2, TEF1, and CYTB). Phylogenetic studies based on one or several genes will probably be insufficient, with different phylogenetic studies producing highly contradictory results (Aime et al., 2006; Schell et al., 2011; Wang et al., 2015; Zhao et al., 2017). By comparison, the compilation of all single-copy genes will improve phylogenetic reconstructions due to increased statistical power (Salichos and Rokas, 2013). Furthermore, there are extreme differences in the life cycles, trophic modes, and genome contents of rust fungi and other fungi (Lorrain et al., 2019; Bakkeren and Szabo, 2020). In general, the more ancient the divergence is, the more diversity it accumulates (O'Meara, 2012). Thus, it is reasonable to place rust fungi as the earliest diverging lineage of the selected fungi.

For phytoparasitic fungi, external nutrient sources are likely to be scarce until successful penetration (Solomon et al., 2003; Divon and Fluhr, 2007). Using mechanical pressure to support hyphal elongation and penetration seems to be an ideal way to adapt to nutrient stress. Vacuoles undergo dramatic expansions during colonization in rust fungi and anther smuts (Chen et al., 2013; Kawamoto et al., 2017), which can produce turgor pressure to lessen the metabolic demands for cytoplasm biosynthesis (Richards et al., 2010). Vacuolar-type H^+^-ATPase (V-ATPase) is responsible for pumping protons into the vacuole, generating membrane potential for solute transport, which is important in the generation of turgor pressure (Smart et al., 1998). Consistently, signatures of convergence related to V-ATPase could be observed. ATPeV0D, which belongs to the V0 subunit of the V-ATPase, plays a role in the coupling of proton transport and ATP hydrolysis (Smith et al., 2005). In ATPeV0D, helix 1 and helix 5 are important domains required for V1Vo assembly and the translocation of protons, respectively (Owegi et al., 2006). The convergence that occurred in these two domains may enhance efficiency in the generation of turgor pressure, reducing energy consumption for hyphal growth and penetration.

The colonization process of phytoparasitic fungi is complex and continuous and requires support from protein synthesis. For this reason, ribosome-associated proteins could play an important role in colonization processes (Wang et al., 2019). We surmised that the sustainability and high efficiency of protein synthesis may facilitate colonization. RP-S27AE is a fusion protein composed of N-terminal ubiquitin and C-terminal ribosomal proteins. The ubiquitin component has a chaperone function, which aids in the correct folding and synthesis of ribosomal proteins (Lacombe et al., 2009). It has been proven that the Ser57Ala mutation occurring in the ubiquitin component can improve ubiquitin homeostasis in yeast (Lee et al., 2017). Hence, convergence may facilitate the sustainability of ribosomal biosynthesis because higher homeostasis could mean a longer working time. The positively selected genes were consistent with the results observed for convergent substitutions. In addition, there were eight genes involved in translation under positive selection in rust fungi and anther smuts. Nonsynonymous mutations occurring in these genes may help phytoparasitic fungi enhance their protein production, reflecting their adaptive evolution.

In rust fungi and anther smuts, secreted proteins are vital in interactions with host plants, and they have been proven to be under selection pressure to undergo nonsynonymous amino acid changes to avoid detection (Aguileta et al., 2010; Hacquard et al., 2012; Sperschneider et al., 2014). However, nonsynonymous amino acid changes may cause protein structural changes that impair folding and modification. To ensure the normal secretion of rapidly evolving secreted proteins, it is not surprising that genes related to the secretion pathway are under positive selection. We found that three genes essential for secretion were under positive selection in rust fungi and anther smuts. Among them, SEC24 promotes the formation of transport vesicles from the endoplasmic reticulum (ER), CANX promotes the accurate folding of glycoproteins entering the secretory pathway, and POMT is responsible for attaching mannose to serine and threonine residues of secreted proteins, modulating their location and function (Gentzsch and Tanner, 1996; Williams, 2006; Wendeler et al., 2007). Taken together, these findings suggest that positive selection on genes results in efficient transport, accurate folding, and precise modification of secreted proteins and thus has a favorable effect on colonization.

The convergent expansion of genes involved in oligopeptide uptake and metabolism could also reflect the adaptive evolution of a phytoparasitic lifestyle. OPTs are believed to translocate oligopeptides from the extracellular environment into the cytosol (Lubkowitz, 2011). Oligopeptides, an important nitrogen source for fungi, are abundant in plants (Becerra-Rodríguez et al., 2020). To adapt to this nutritional environment, OPTs were likely expanded in rust fungi and anther smuts. Moreover, some OPTs have been demonstrated to be significantly upregulated during infection by rust fungi and anther smuts (Duplessis et al., 2011; Toh et al., 2017). The expansion and high expression level of OPTs may improve the capacity for oligopeptide uptake in rust fungi and anther smuts, which benefits fungal growth and colonization. Moreover, the expansion of vacuolar proteases could support the above hypothesis because larger numbers of vacuolar proteases may be better for the degradation and recycling of oligopeptides.

## Conclusion

This study is the first to show common adaptive mechanisms underlying phytoparasitic lifestyles in rust fungi and anther smuts. Signatures of convergence were identified based on convergent substitutions, positive selection, and gene gains or losses. A total of two convergent genes, 51 positively selected genes, and two expanded orthogroups were identified. These convergence events suggest four adaptive mechanisms underlying a phytoparasitic lifestyle: 1) reducing the metabolic demand for hyphal growth and penetration at the pre-penetration stage, 2) maintaining the efficiency of protein synthesis during colonization, 3) ensuring the normal secretion of rapidly evolving secreted proteins, and 4) improving the capacity for oligopeptide metabolism. However, existing methods mainly focus on strict single-copy genes, which account for a small portion of all genes in the selected fungi. For this reason, a small amount of evidence for convergence was found in our study. With the development of new methods, we expect that more evidence for convergence between rust fungi and anther smuts will be discovered in the future.

## Data Availability

The datasets presented in this study can be found in online repositories. The names of the repository/repositories and accession number(s) can be found in the article/Supplementary Material.

## Supplementary Material

Supplementary Figure 1 Flow chart describing the main steps of convergence analyses.

Supplementary Figure 2 The divergence times of 19 selected fungi.

Supplementary Table 1 Detailed information on the selected fungi.

Supplementary Table 2 General statistics about orthologous gene groups.

Supplementary Table 3 The ID of genes for each orthogroup.

Supplementary Table 4 Convergent analyses in rust fungi and anther smuts.

Supplementary Table 5 Selective pressure analyses in rust fungi and anther smuts.

Supplementary Table 6 The number of genes for each orthogroup.

Supplementary File 1 The results of genome annotation, FASTA format sequences for RAxML-NG, FASTA format sequences for conv_cal, and PML format sequences for CodeML.
