# Supplementary figures and images for: Convergence Analysis of Rust Fungi and Anther Smuts Reveals Their Common Molecular Adaptation to a Phytoparasitic Lifestyle
Source: Front Genet. 2022 Apr 8;13:863617. doi: 10.3389/fgene.2022.863617 (PMC9023891; doi:10.3389/fgene.2022.863617)

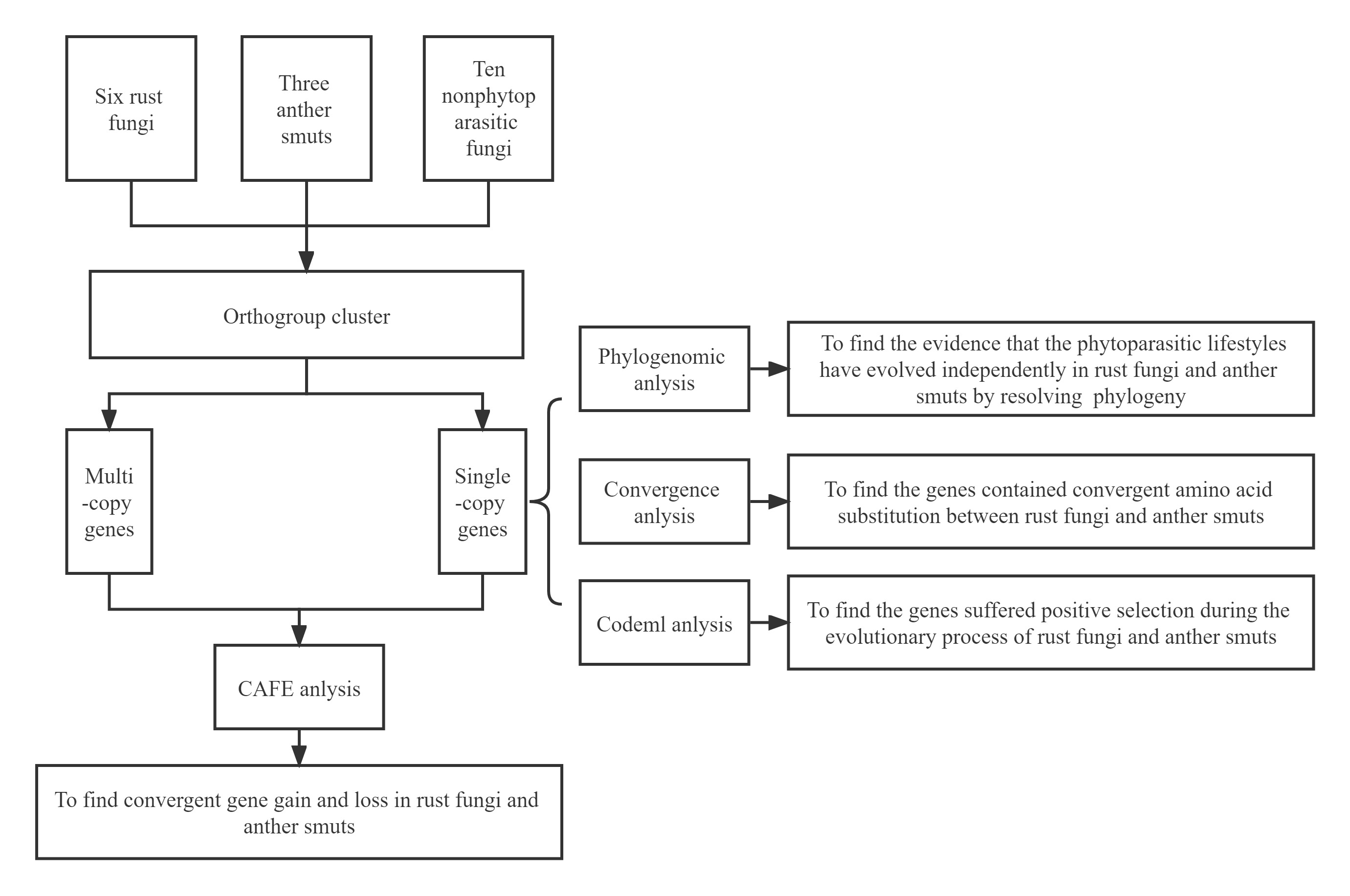

Supplement: Supplementary file 3 [file Image1.JPEG]

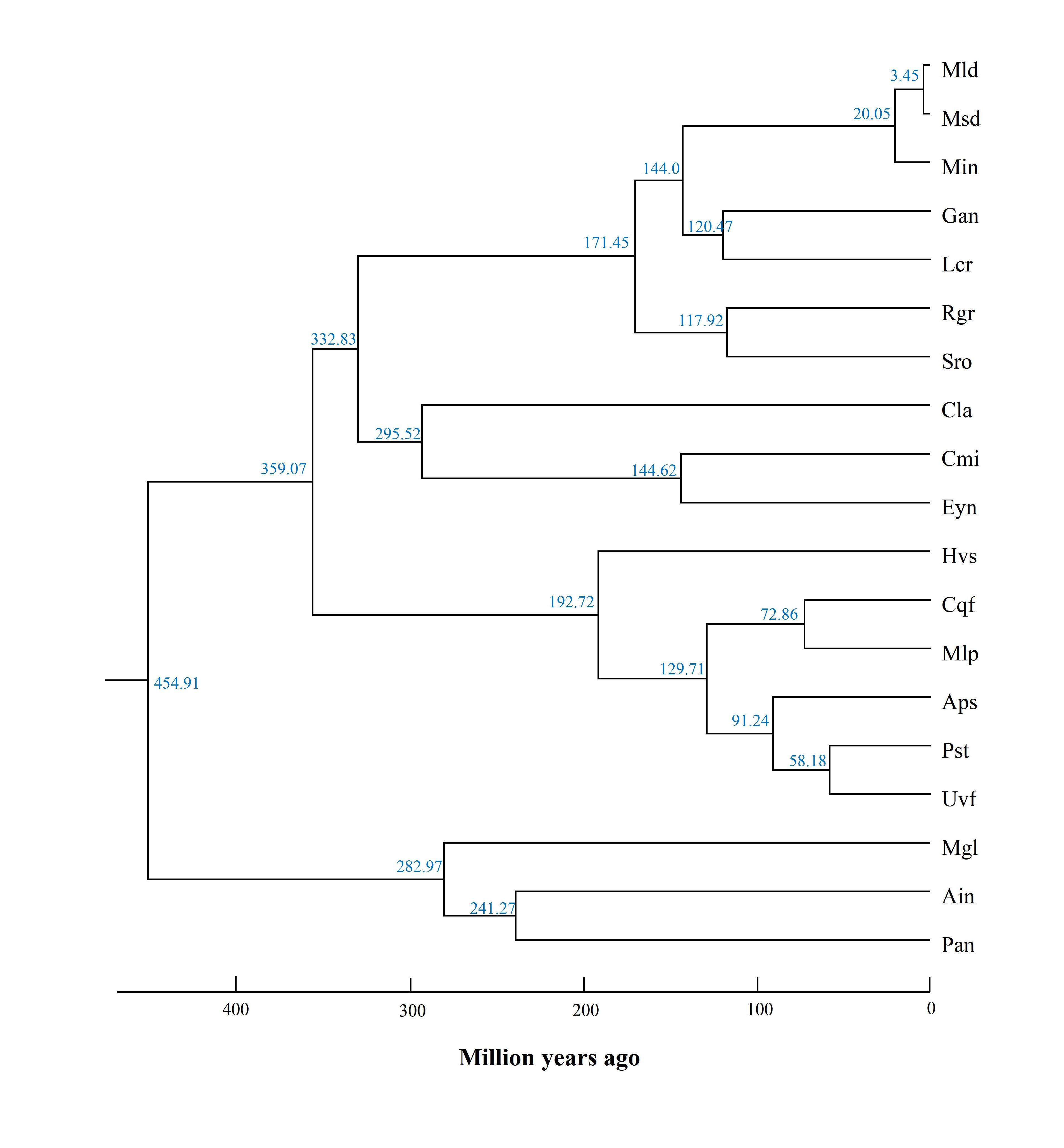

Supplement: Supplementary file 5 [file Image2.JPEG]
